# Rapid and automated risk stratification by determination of the aortic stiffness in healthy subjects and subjects with cardiovascular disease

**DOI:** 10.1371/journal.pone.0216538

**Published:** 2019-05-13

**Authors:** Julia Lortz, Lennard Halfmann, Amelie Burghardt, Martin Steinmetz, Tobias Radecke, Rolf Alexander Jánosi, Tienush Rassaf, Christos Rammos

**Affiliations:** Department of Cardiology and Vascular Medicine, West German Heart and Vascular Center Essen, University of Duisburg-Essen, Essen, Germany; Universitatsklinikum Wurzburg, GERMANY

## Abstract

**Background:**

Aortic stiffness is an independent predictor of cardiovascular morbidity and mortality; thus, simple, rapid and preferably automated techniques are indispensable for pursuing a global risk stratification approach. We present an oscillometric technique for determination of the carotid-femoral pulse wave velocity (cfPWV), including the diagnostic accuracy, sensitivity and specificity, with emphasis on the training curve and procedural duration.

**Methods:**

In a single-centre crossover study, we evaluated subjects free of known cardiovascular disease (CVD), subjects with CVD and a subgroup of subjects with peripheral artery disease (PAD) in terms of ankle-brachial index (ABI) and PWV measurements determined by oscillometry compared to tonometry. Pearson’s correlation analysis was used to assess the relationship of the PWV measurements determined by both methods. Moreover, the time and cost of the examinations were compared.

**Results:**

A total of 176 study subjects underwent assessments to obtain oscillometric and tonometric PWV measurements. The CVD-free subjects (*n* = 59) were younger (60.4±15.6 vs. 67.5±12.9 years, *p* = 0.003) than the subjects with CVD (*n* = 117). The PWV measurements showed significant correlations in CVD-free subjects (*r* = 0.797, *p*<0.001), in subjects with CVD (*r* = 0.817, *p*<0.001) and in the subgroup of subjects with PAD (*r* = 0.807, *p*<0.001). The examination duration was shorter for the oscillometric method than the tonometric method (4.4±0.5 vs. 9.2±0.8 min, *p*<0.001).

**Conclusion:**

Using a simple and rapid automated oscillometric method, we achieved good diagnostic accuracy for the determination of aortic stiffness through the PWV in both subjects with and without CVD. This method might be helpful in daily practice in terms of saving time and reducing procedural complexity for screening for cardiovascular morbidities and vascular damage in cases of atherosclerosis.

## Introduction

Endothelial dysfunction, atherogenic alterations and vascular stiffness are key in the development and perpetuation of cardiovascular disease. While most atherosclerotic lesions are only visible though invasive and non-invasive angiography, the earliest phenomenon of atherosclerotic cardiovascular disease (CVD) indicating inappropriate regulation of the vascular tone is endothelial dysfunction [1]. However, the determination of endothelial dysfunction by flow-mediated dilation is time consuming, highly dependent on an operator’s expertise, and influenced by a number of environmental factors [2,3].

Increased aortic stiffness reflects advanced vascular alterations associated with ageing, CVD, diabetes and renal disease and is independently associated with higher rates of all-cause cardiovascular morbidity and mortality [4–7].

The non-invasive detection of early vascular alterations and the associated cardiovascular risk has gained increasing impact in everyday clinical practice [8]. The carotid-femoral pulse wave velocity (cfPWV) is the current gold standard for the non-invasive assessment of aortic stiffness [9], and emerging evidence supports that central haemodynamics might be superior to brachial blood pressure in assessing cardiovascular risk and evaluating target organ damage [10–12]. Tonometry is widely established for the clinical assessment of cfPWV [7,13]. In this method, applanation tonometry is used to determine the cfPWV through recordings from the carotid and femoral arteries. However, the elaborate handling required and the extended examination duration limits its widespread use. Thus, rapid and automated tools for risk stratification by the determination of aortic stiffness are needed.

Another method is the oscillometric detection of pulse waves during cuff occlusion of the brachial artery. This method is characterized by an increased ease of use, lower operator skill requirements and a shorter examination duration and might therefore simplify everyday practice.

The aim of this study was to determine the diagnostic accuracy of an automated and rapid oscillometric device for determination of the cfPWV compared with the clinically validated and widely accepted tonometric gold standard.

## Methods

### Study design and participants

In a single-centre crossover study, we included study subjects from the outpatient clinic of the Department of Cardiology and Vascular Medicine at the West German Heart and Vascular Center, University Hospital of Essen, Germany.

The study subjects were divided into two groups: 1.) Study subjects without any previous history of cardiovascular disease, renal disease, or diabetes and with an ankle-brachial index (ABI) > 0.9 were considered free of CVD. 2.) Study subjects with CVD were substratified into groups of subjects with coronary artery disease (CAD) and peripheral arterial disease (PAD). Criteria for subjects with CAD were known previous coronary interventions or a history of coronary artery bypass grafting. Subjects with PAD were defined as having at least one of the following: a history of surgical or interventional revascularisation and an impaired walking distance of < 200 m or a walking distance > 200 m but an ABI < 0.9.

The exclusion criteria were severe heart failure (NYHA III-IV; classification for heart failure defined by the New York Heart Association), severe valvular heart disease and/or significant cardiac arrhythmias, including atrial fibrillation and frequent ventricular ectopic beats, and necrosis or ulceration at any measurement location.

All vascular stiffness measurements were performed according to the international guidelines [14] and by the same investigator. The standard measurement approach was always performed in the same quiet room at a consistent controlled temperature and after a resting period of at least 20 minutes. The same examination sequence was maintained for all study subjects. To obtain reliable measurements, the patients were asked not to speak and to lie quietly from cuff inflation to cuff deflation.

The study was approved by the local ethics committee of the University of Duisburg-Essen and was conducted in accordance with the principles of the Declaration of Helsinki. The records of the study subjects were de-identified and analysed anonymously. Each subject provided oral or written consent to participate in the study.

### Ankle-brachial index

The ABI is a useful tool for the diagnosis and surveillance of lower extremity artery disease and serves as a strong marker of generalized atherosclerosis and cardiovascular risk. An ABI ≤0.90 is associated with a 2- to 3-fold increased risk of total and CV death, on average [15]. The ABI was assessed according to the current guidelines [16]. In brief, the systolic blood pressure was assessed using a pneumatic cuff and Doppler measurements at the brachial, dorsalis pedis and posterior tibial arteries (left and right). The ABI was determined by calculating the highest ratio of the highest systolic pressure at the brachial artery to the systolic pressure at the ankle:

$$Right\;ABI\;=\;\frac{A}{B}$$

A = Highest systolic pressure right ankle

B = Highest systolic pressure of both arms

The measurements were performed under the conditions of a standardized room temperature (approximately 25°C) after 15 minutes of acclimatization.

### Carotid-femoral pulse wave velocity

The carotid-femoral (cf)PWV was measured by applanation tonometry using a SphygmoCor XCEL system (AtCor Medical, Australia), as previously described [7,13,17,18]. The indirect measures of arterial stiffness and central haemodynamics were obtained with the subject in a supine position. Carotid arterial pressure waveforms were obtained by applanation tonometry. Applanation tonometry has been validated to yield a precise assessment of intraarterial pressures by comparison with simultaneous invasive pressure recordings [19].

The PWV was calculated from sequential recordings of electrocardiogram-referenced carotid and femoral pressure waveforms obtained by tonometry using the SphygmoCor device and transducer. For the assessment of the wave transit time, the dedicated built-in SphygmoCor software (SphygmoCor XCEL Software Version: 1.3.2.13) was used to determine the distance between the carotid and femoral sites considering the distance between each artery location and the sternal notch. The R wave of a simultaneously recorded electrocardiogram served as a reference frame, as previously described [7,9].

### Oscillometric pulse wave velocity

In contrast to applanation tonometry, the automated oscillometric method (Boso ABI system 100 PWV, Bosch & Sohn, Germany) does not require exact positioning of the Doppler transducer at the carotid artery and arteries to assess the pulse wave since it works using four blood pressure cuffs. As such, the exact measurements between the two Doppler points (carotid and femoral arteries) are omitted.

Blood pressure cuffs were wrapped around both upper arms at the brachial arteries and both ankles above the tibial arteries. The cuffs were connected to the Boso device, including an oscillometric pressure sensor that determines the blood pressure volume waveform from the brachial and tibial arteries simultaneously. The Boso ABI system works by analysing the oscillations transferred through the generated pulse wave to the pressure cuff.

**Fig 1:**
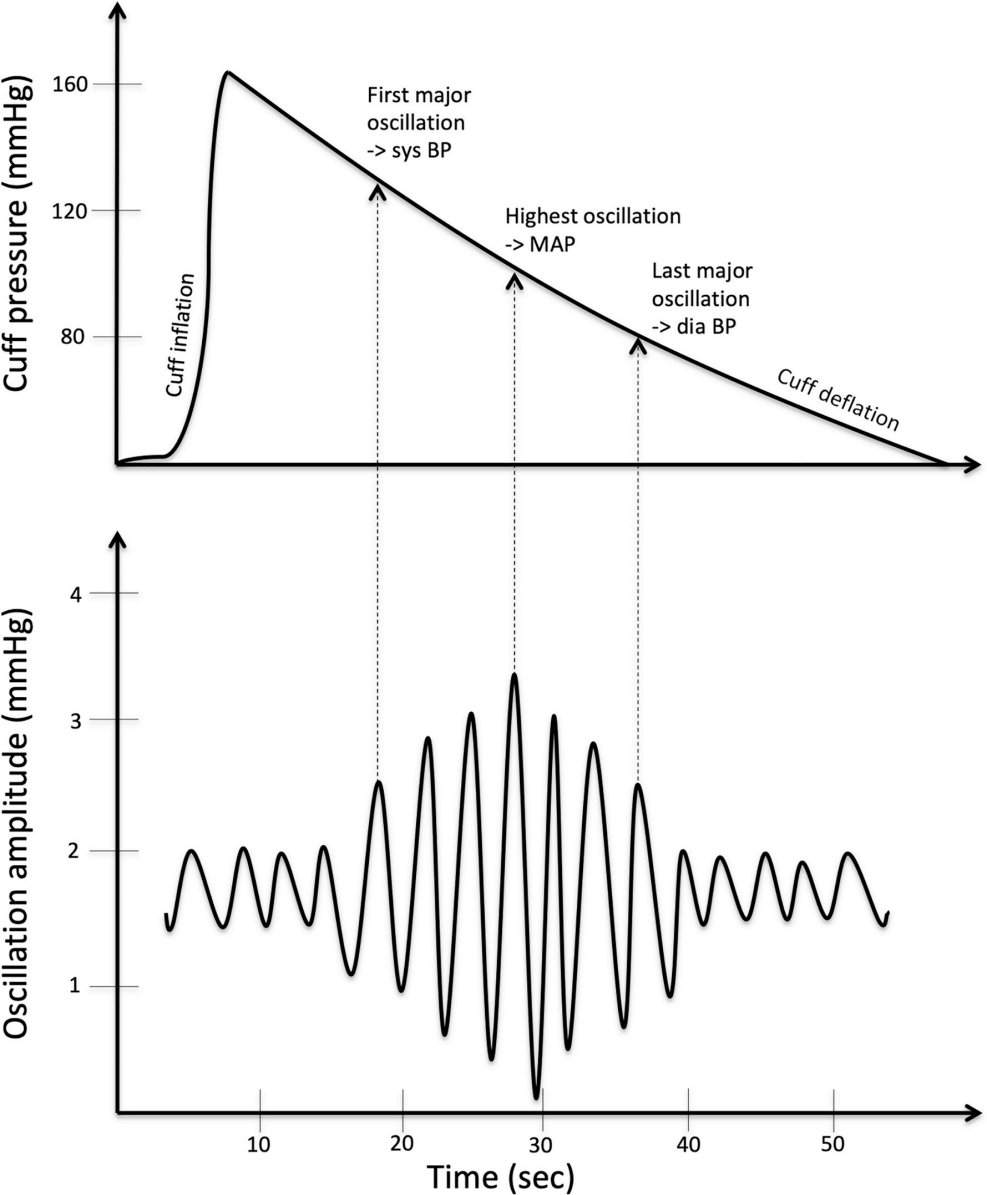
The oscillation pressure pattern reflects typical deduction points in blood pressure (BP) measurements. With the first major oscillation marking the systolic (sys) BP, the amplitude further rises until the maximum peak (mean arterial pressure, MAP). The following decrease ends with the last major oscillation amplitude as the diastolic (dia) BP point.

In detail, the oscillometric method uses the detection of an arterial pressure pulsation in the cuff. By the simultaneous inflation of the cuff, the blood flow is stopped for a short time, and the sole pulsation of the artery can be assessed. The oscillometer measures the magnitude of the pressure oscillation as the cuff is deflated. Routinely, a suprasystolic blood pressure of more than 30 mmHg above the expected systolic blood pressure is obtained. The oscillation amplitude increases in the opposite direction as the cuff pressure declines until the peak amplitude that marks the mean arterial pressure. A further decrease in the cuff pressure leads to a decrease in the oscillation amplitude (Fig 1) [20, 21]. The ABI measurements for each leg were calculated by the software on the basis of the greatest systolic arm pressure. Through simultaneous cuff inflation, in addition to the systolic and diastolic blood pressure, this method also provides information regarding the pulse transit time between the upper and lower cuffs. The determination of the pulse transit time is necessary for the assessment of the baPWV and further calculation of the cfPWV. A detailed description of the PWV measurement using the oscillometric technique, including the underlying regression analysis leading to the following equation, was previously performed by Naidu et al. [21] in healthy subjects:

cfPWV=0.833xbaPWV-2.33(m/s)

In brief, the formula was derived through the recording of waveforms from the brachial and ankle segments. The underlying calculation of the PWV considers the distance covered by the pulse wave in relation to the pulse transit time:

$$PWV\;=\;\frac{D}{PTT}(m/s)$$

D = Distance

PTT = Pulse transit time

**Fig 2:**
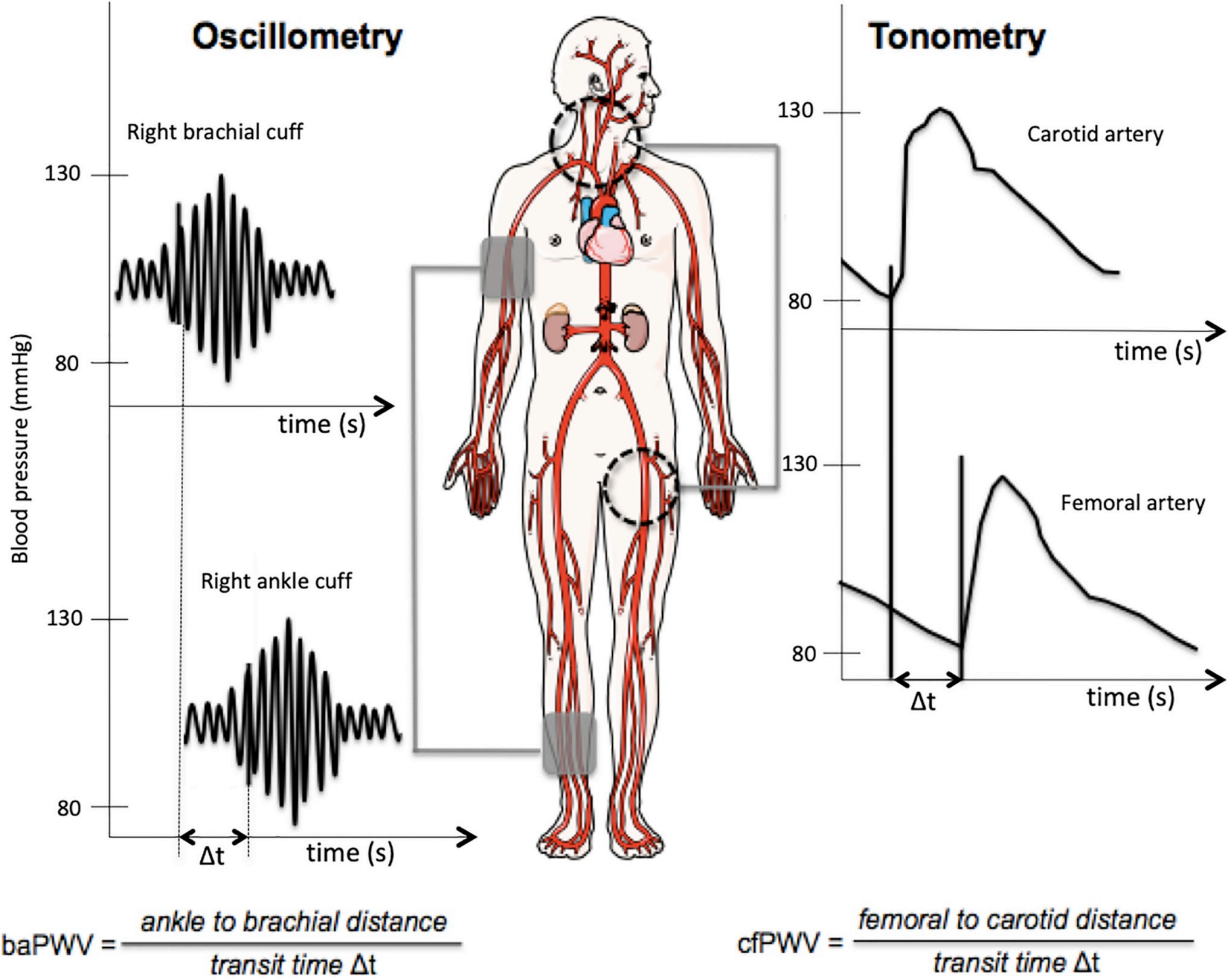
The oscillometric device assesses the brachial-ankle pulse wave velocity (baPWV) through the measurement of the transit time between the brachial artery and tibial artery through the oscillometric amplitude. The determination of the carotid-femoral (cf) PWV results from the following calculation: cfPWV = 0.833 x baPWV -2.333 (m/s). The applanation tonometry device uses the tonometric technique with direct measurement of the cfPWV.

The covered distances were directly superficially measured using a measuring tape. The pulse transit time was assessed by measuring from the R wave to the maximum pressure gradient as the pressure wave arrival marker (Fig 2). The cfPWV was estimated from the composite baPWV after averaging the left and right baPWV. A regression analysis of the baPWV and cfPWV yielded the following formula: cfPWV = 0.833 x baPWV–2.333 (m/s). This formula was derived in a study of healthy subjects to determine the interperiod and interobserver reproducibility and was validated in studies of patients with cardiovascular disease [22] and patients with type 2 diabetes [23].

The assessed patient data, including height, were entered into the dedicated software of the Boso ABI system (Boso profil-manager XD, Bosch & Sohn, Germany).

To gain insight into the time and cost effectiveness of both methods, the time for twenty measurements was recorded.

#### Doppler-assisted ABI measurements

ABI measurements were obtained using a Doppler probe on the posterior tibial and anterior tibial artery locations, as previously described [22,24]. According to current guidelines, the highest value was used for the calculations and divided by the highest systolic brachial Doppler pressure [16]. In brief, the cuff was inflated to suprasystolic pressure (i.e., >30 mmHg above the expected systolic pressure) and deflated slowly until a flow signal was detected by Doppler over the anterior tibial artery and posterior tibial artery, respectively. The brachial artery systolic pressure was assessed similarly on both upper extremities. The higher systolic pressure was used for the ABI calculations.

### Biochemical analyses

Blood was drawn for routine clinical analyses, and the Institute of Clinical Chemistry and Laboratory Diagnostics, University Hospital Essen, performed all analyses unless otherwise noted.

### Statistical analysis

Continuous variables are presented as the mean ± standard deviation, while categorical variables are presented as frequencies or percentages.

After checking the data in terms of distribution normality, we compared differences in the patient characteristics using a 2-sided t-test for continuous variables and Fischer’s exact test for binary variables. Spearman’s rank correlation coefficient, *r*, was computed to assess the relationships between variables. The Bland–Altman test was used to evaluate the variability between the two techniques.

Linear regression showed that the cfPWV_tono_ had a significant ability to predict the cfPWV_osc_, F(1, 169) = 326.6, p < 0.0005, and the measured cfPWV_tono_ accounted for 12.9% of the explained variability in the cfPWV assessment. The regression equation was as follows: predicted cfPWV_osc_ = 2.397 + 0.747 x (cfPWV_tono_) (m/s).

Values of *p* < 0.05 were considered statistically significant.

All data and statistical analyses were performed using SPSS 24 (Chicago, IL, USA) for Mac and Microsoft Excel 2011 for Mac.

## Results

### Study population

A total of 176 study subjects were included. The study subjects were aged from 21 to 90 years and were predominantly male (*n* = 125, 71%). One-third (*n* = 59) of the subjects did not have any relevant cardiovascular disease and were considered free of CVD. Two-thirds (*n* = 117) of the subjects were diagnosed with CAD or PAD. Eighty-seven study subjects were diagnosed with PAD. Forty-six of the subjects with PAD had a limited walking distance of < 200 m classified as Fontaine IIb, and 18 subjects reported pain at rest (Fontaine III). Twenty-two subjects were diagnosed with PAD, but without any walking distance limitations (Fontaine I/II). The relevant study subject characteristics are shown in Table 1.

**Table 1:** Study subject characteristics.

	CVD-free	CVD	*p* value
	*n* = 59	*n* = 117	
Age (years)	60.4± 15.6	67.5 ± 12.9	0.003*
Men, n (%)	43 (73)	82 (71)	0.700
Weight (kg)	79.6 ± 14.7	83.1 ± 17.7	0.193
Height (m)	1.72 ± 9.2	1.74 ± 9.2	0.195
BMI (kg/m^2^)	27 ± 4.8	27.2 ± 5.2	0.736
SBP (mmHg)	136.8 ± 25.3	127 ± 30	0.069
DBP (mmHg)	73.6 ± 13	76.3 ± 13.4	0.290
*Biochemical analysis*			
Plasma hsCRP (mg/l)	0.24 ± 0.21^a^	0.33 ± 0.24^b^	0.030*
Total cholesterol (mg/dl)	182 ± 49.4^a^	174 ± 38.9^b^	0.267
HDL (mg/dl)	49.4 ± 16.3^a^	48.2 ± 17.1^b^	0.681
LDL (mg/dl)	113 ± 41.7^a^	106.6 ± 34.7^b^	0.315
Triglycerides (mg/dl)	160.7 ± 90.8^a^	156.2 ± 99.2^b^	0.781
Nt-proBNP (pg/ml)	1,046 ± 3,036^a^	4,423 ± 32,127^b^	0.439

CVD, cardiovascular disease; BMI, body mass index; SBP, systolic blood pressure; DBP, diastolic blood pressure; hsCRP, high-sensitivity C-reactive protein; HDL, high-density lipoprotein; LDL, low-density lipoprotein. Data are presented as the mean ± standard deviation or *n* (%).

* indicates significance.

^a^ *n* = 55,

^b^ *n* = 99.

### Comparison of the ankle-brachial index

**Fig 3:**
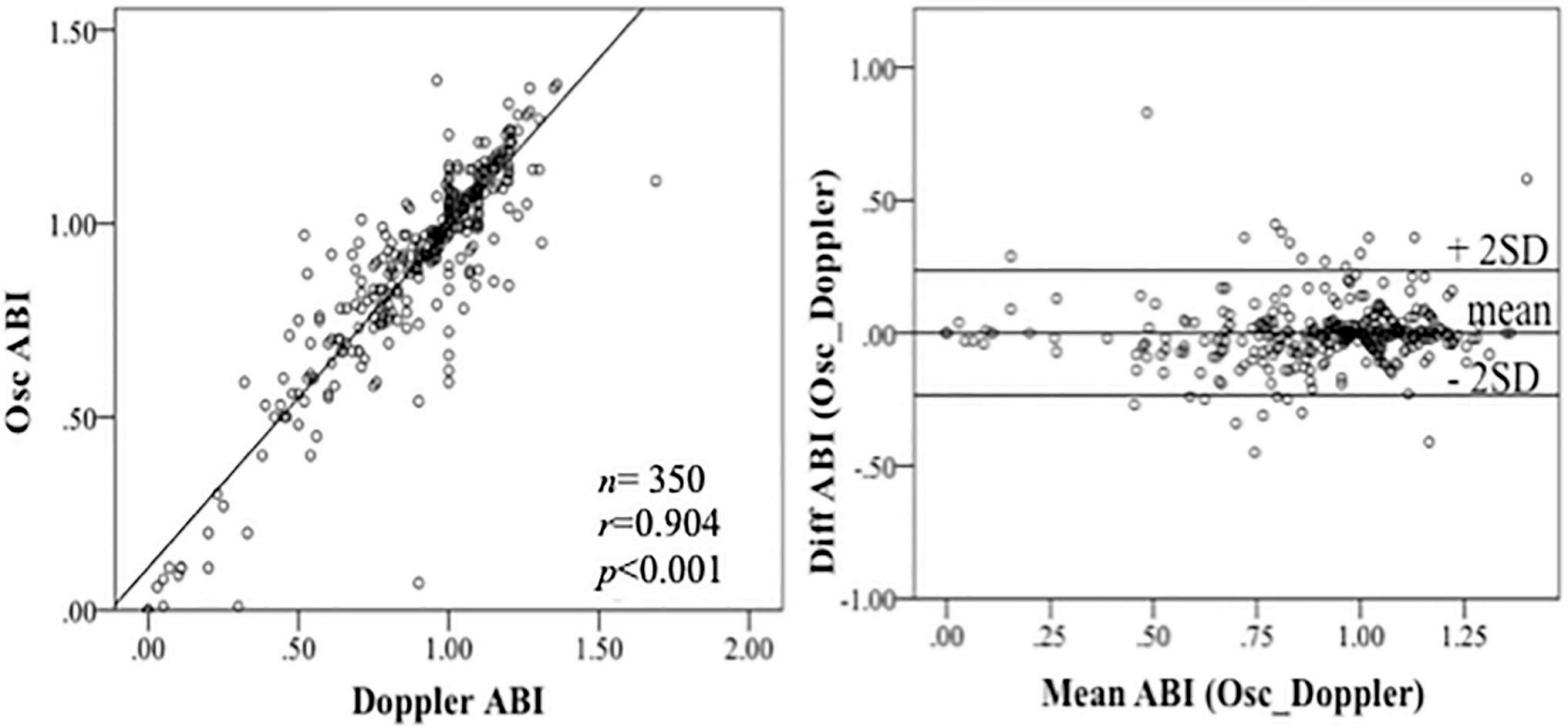
Correlation of the ankle-brachial index (ABI) determined by Doppler and oscillometry in the overall population. (A) High correlation between the ABI values determined by Doppler and oscillometry. (B) Bland–Altman plot of the agreement between the ABI values determined by Doppler and oscillometry.

The ABI assessment was performed using a Doppler probe and oscillometry. From a total of 176 study subjects, we obtained 350 ABI values with a mean of 0.9 ± 0.28 for the Doppler method and 0.9 ± 0.26 for the oscillometry. The overall correlation was highly significant (*r* = 0.904, *p*<0.001, Fig 3). The subgroup analysis showed a good correlation in the CVD-free subjects (*r* = 0.913, *p*<0.001) and in the subjects with CVD (*r* = 0.881, *p*<0.001).

### Comparison of the carotid-femoral pulse wave velocity

**Fig 4:**
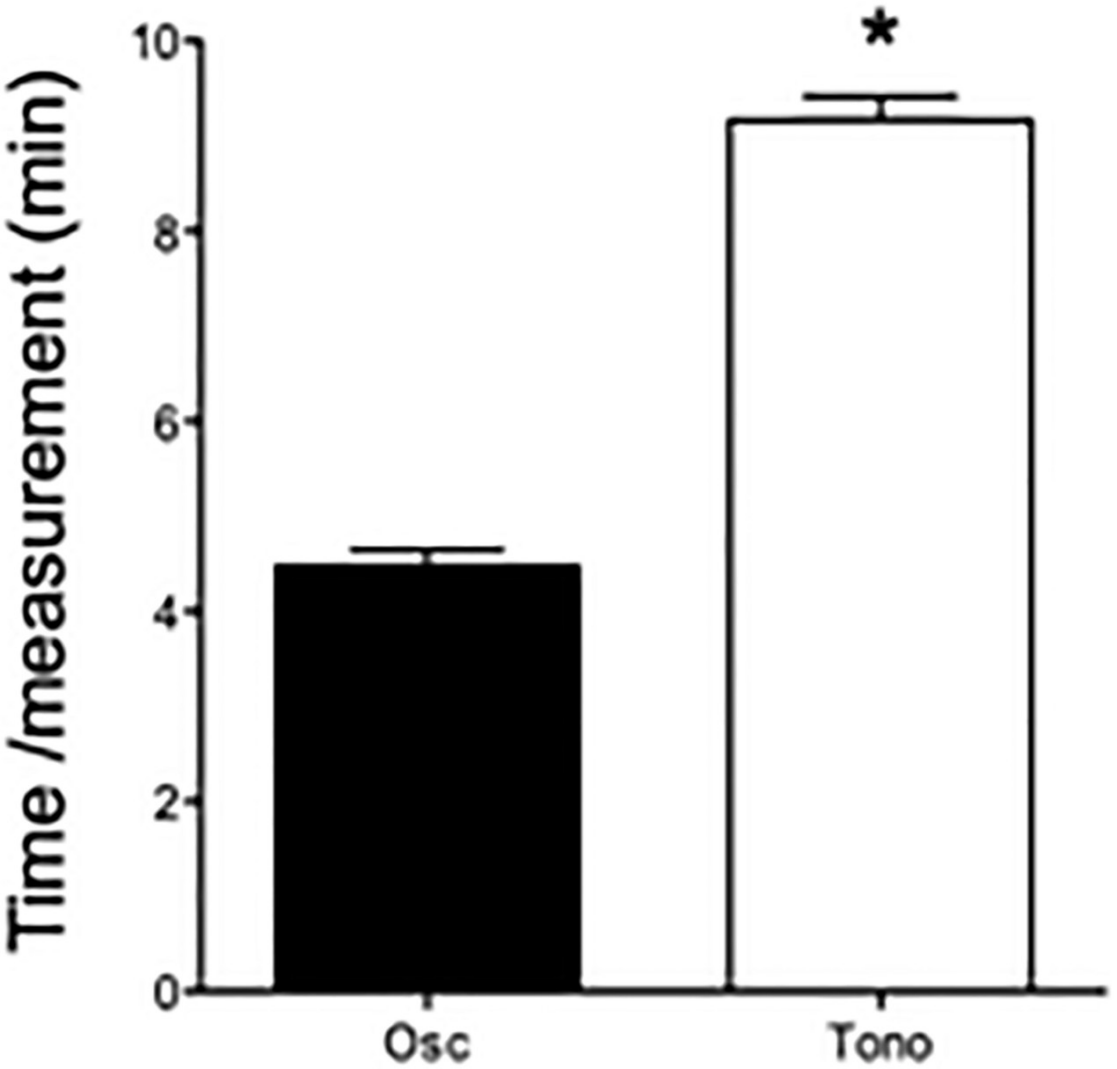
The oscillometric method (osc) shows advantages in terms of the time required per examination compared to the tonometric method (tono). Shown is the required time per pulse wave velocity measurements, including the mean and standard deviation for 20 measurements each.

The PWV measurements were subsequently obtained by applanation tonometry and the oscillometric technique in one session. The time was recorded for twenty measurements. Applanation tonometry required more than twice the time to assess the cfPWV (oscillometry 4.4 ± 0.5 min vs. tonometry 9.2 ± 0.8 min, *p*<0.001, Fig 4).

#### Overall population

**Fig 5:**
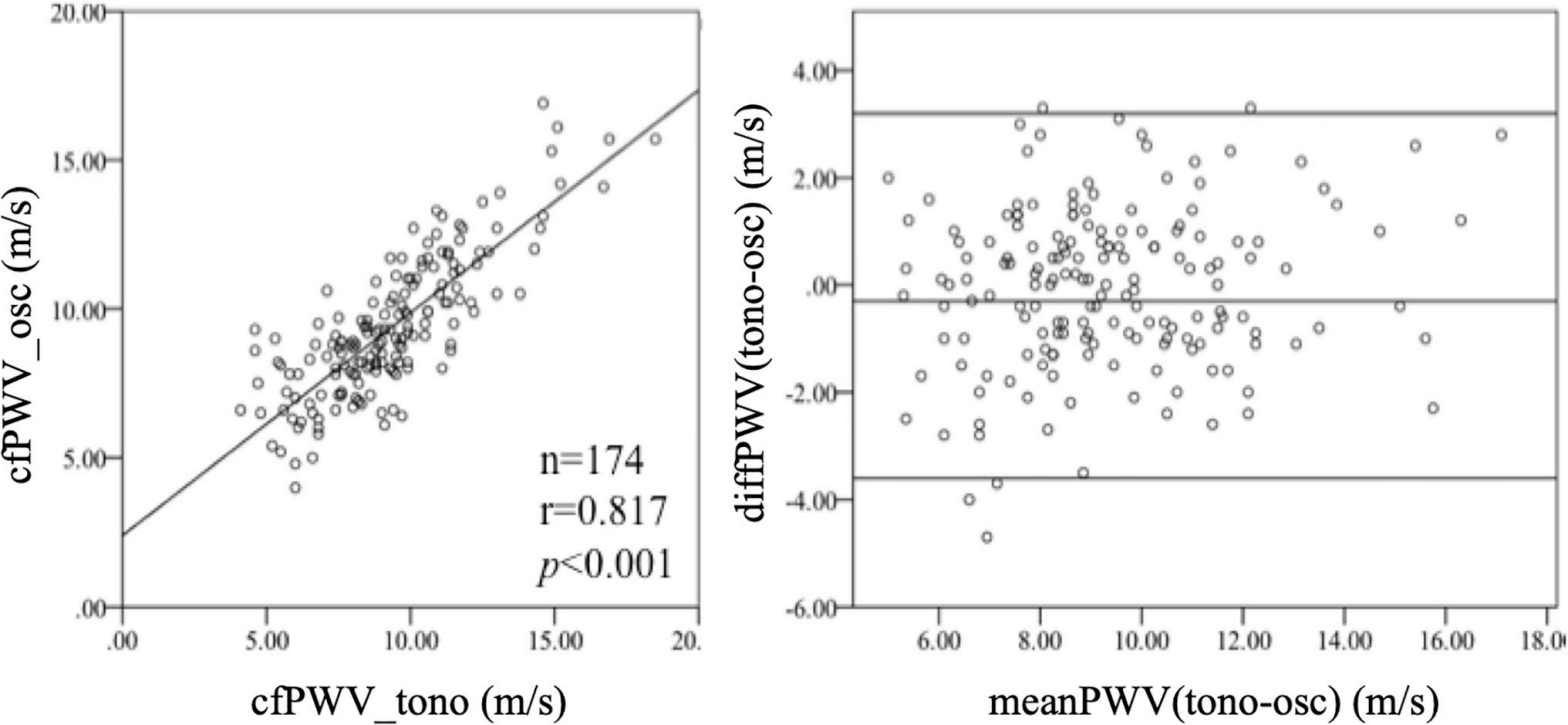
The oscillometric (osc) and tonometric (tono) assessments of the carotid-femoral pulse wave velocity (cfPWV) show a good overall correlation. The left graph shows the correlation between the cfPWV determined by both methods. The right graph shows the corresponding Bland–Altman plot of the agreement between tonometric and oscillometric assessments of the cfPWV.

Further PWV measurements were obtained from a total of 176 study subjects. No significant differences (*p* = 0.256) were observed between the transit time measured by the tonometric method (60.7 ± 18 ms) and the oscillometric method (61.8 ± 17.2 ms, *p* = ns), yielding a good correlation (*r* = 0.744, *p*<0.001). Comparing the respective tonometric and oscillometric methods revealed highly significant correlations regarding the cfPWV. The mean cfPWV_tono_ (9.3 ± 2.6 m/s) and the mean cfPWV_osc_ (9.4 ± 2.3 m/s) did not differ (*p* = 0.742) and were highly correlated (*r* = 0.817, *p*<0.001, Fig 5).

Linear regression was applied to understand the relation of the tonometric cfPWV with the oscillometric cfPWV. To assess linearity, a scatterplot of the cfPWV_osc_ against the cfPWV_tono_ with a superimposed regression line was created. Visual inspection of these two plots indicated a linear relationship between the variables. The residuals showed both homoscedasticity and normality, as well as independence, as indicated by a Durbin–Watson statistic of 1.775.

The prediction equation was as follows: cfPWV_osc_ = 2.397 + 0.747 x (cfPWV_tono_) m/sec. The CfPWV_tono_ showed a significant ability to predict the cfPWV_osc_, F(1, 169) = 326.6, p < 0.0005, accounting for 12.9% of the variation in the cfPWV_osc_ values. Predictions were made to determine the cfPWV_osc_ for the measured cfPWV_tono_ values. The cfPWV_osc_ was predicted as 0.812 (95% CI, 0.66 to 0.83) m/sec.

#### Subjects without CVD versus subjects with CVD

**Fig 6:**
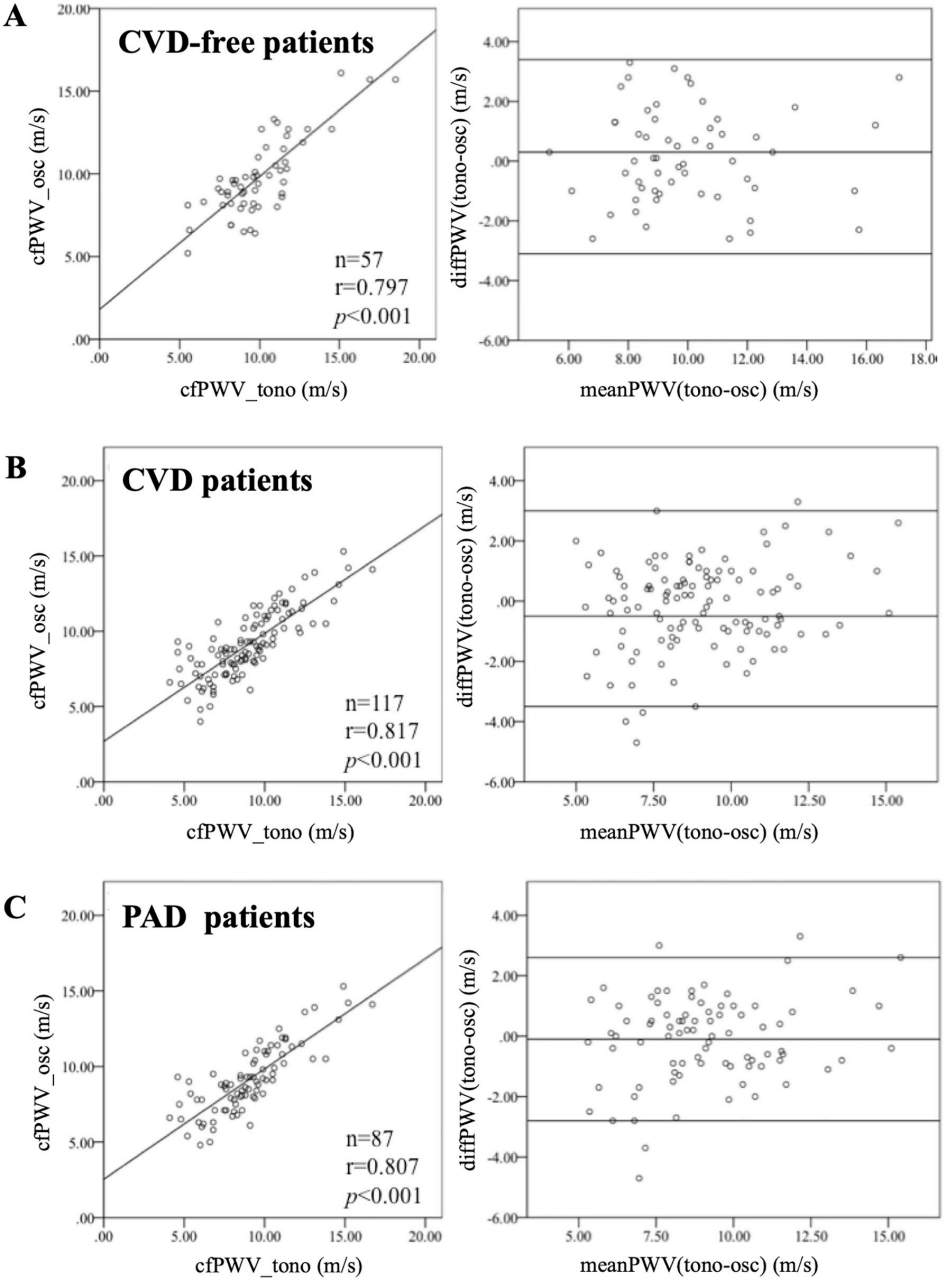
The oscillometric (osc) and tonometric (tono) assessments of the carotid-femoral pulse wave velocity (cfPWV) show a good correlation in the subgroups. The results of the subgroup analysis of subjects without cardiovascular disease (A, CVD-free), with cardiovascular disease (B, CVD) and with peripheral artery disease (C, PAD) are demonstrated as Spearman’s rank correlation coefficients (left) and the corresponding Bland–Altman plots (right).

A good correlation of the PWV determined by both techniques was found in both subjects with and without CVD. In the subjects without CVD (*n* = 57), the cfPWV_tono_ (8.7 ± 2.2 m/s) and cfPWV_osc_ (8.4 ± 1.9 m/s, *p* = 0.181) values were comparable and showed a good correlation (*r* = 0.797, *p*<0.001, Fig 6A). These findings were corroborated in the subjects with CVD (*n* = 117): cfPWV_tono_, 9.7 ± 2.7 m/s; cfPWV_osc_, 9.8 ± 2.3 m/s, *p* = 0.203; *r* = 0.817, *p*<0.001, Fig 6B). A further subanalysis of the subjects with PAD was performed to test the reliability of the oscillometric technique since this method includes the entire vascular system of the lower limbs, in contrast to applanation tonometry. This subgroup (*n* = 87) revealed the highest cfPWV. The cfPWV_tono_ (10.3 ± 2.6 m/s) was comparable to the cfPWV_osc_ (10.3 ± 2.3 m/s, *p* = 0.835), with a significant correlation (*r* = 0.807, *p*<0.001, Fig 6C).

#### Diabetpic subjects versus non-diabetic subjects

One-third (*n* = 55, 32%) of the study subjects were diabetic and being treated accordingly (i.e., with oral antidiabetic drugs and/or insulin). The cfPWV_tono_ (9.3 ± 2.7 m/s) was comparable to the cfPWV_osc_ (9.3 ± 2.2 m/s, *p* = 0.916) and showed a good correlation (*r* = 0.787, *p*<0.001). The same relationship was detected in the non-diabetic subjects (*n* = 120): the cfPWV_tono_ (9.3 ± 2.5 m/s) was comparable to the cfPWV_osc_ (9.4 ± 2.4 m/s, *p* = 0.616) and showed a good correlation (*r* = 0.833, *p*<0.001).

Analysis of the overall sensitivity and specificity of the aortic stiffness determined by the oscillometric method for risk stratification yielded 93% sensitivity and 84% specificity.

## Discussion

We present an accurate and reliable method for risk stratification through determination of the aortic stiffness in subjects without CVD, subjects with CAD and subjects with PAD using a simple and rapid automated oscillometric device.

To provide an effective risk stratification tool for the broad spectrum of healthcare providers, several challenges must be overcome: the tool should not only produce accurate and valid results but also be easy to use and efficient. In the current study, we tested the oscillometric PWV assessment against the current gold standard of applanation tonometry in terms of these requirements and found favourable results. Although the oscillometric measurement of the PWV is based on an indirect detection method involving the brachial artery and ankle (baPWV), the height-adjusted estimation of the cfPWV was found to be highly accurate. Different diagnostic methods have been validated for cfPWV measurements, as previously described in several studies [11,17,18,25–27]. However, until now, the increased usability of the oscillometric cfPWV assessment with reduced costs has not been investigated.

Epidemiological studies have demonstrated the independent predictive value of the aortic stiffness for CV events. Conclusively, assessment of the PWV is recommended for screening by the current guidelines of the European Society of Hypertension and Cardiology [12].

Various commercialized machines appear to measure the PWV non-invasively, but their market entry is still limited due to the high costs and frequently elaborate handling techniques required. Among some other applied methods, tonometry has been the most widely used and accepted in clinical studies [13,25].

In addition to this accepted concept of tonometric pulse wave assessment, the clinical validation of the oscillometric reference technique in this study is based on the predictive implication of the cfPWV measurement. Our results highlight the correlation between a rapid oscillometric method and the clinically validated tonometric method for determination of the cfPWV and present a highly cost-effective method for assessing the cfPWV using an oscillometric device. In the present study, we found a highly significant correlation between the oscillometric determination of arterial stiffness and wave reflection, determined as the cfPWV, and the conventional tonometric determination of arterial stiffness. Clearly, the strength of this study is the good correlation between the two methods in both subjects without CVD and a large cohort of subjects with CVD, the method could even differentiate between subjects with CAD and PAD. Notably, while no differences were found between the patients with and without CVD in terms of blood pressure, lipid profile or BMI, we found a significant difference in age and the inflammatory profile, as indicated by the hsCRP level. However, a direct relationship between inflammation-associated vascular disorders and stiffness cannot be determined based on the presented data. The subgroup analysis of the subjects with PAD is of particular interest because assessment of the PWV between the brachial artery and the ankle (baPWV) is used to estimate the PWV between the carotid and femoral arteries (cfPWV). Importantly, this assessment was found to be valid in both subjects with and without CVD. The presence of atherosclerosis within the arteries of the lower limbs might result in measurement inaccuracy, which this might be a confounder when analysing oscillometric signals, especially those derived from the calf. Typically, this could be the case in subjects with established PAD and an ankle-brachial index <0.9. Herein, we provide the first evidence that even in patients with established PAD, automated determination of the pulse wave velocity is feasible and accurate, which is clearly a strength of the study. Even in subjects with PAD, the accuracy of the oscillometric PWV assessment was high.

Measurement of the cfPWV by applanation tonometry using the SphygmoCor XCEL system requires the measurement of the distance between the jugular and carotid arteries to be adjusted for the proper aortic length. Tonometric measurement of the carotid pulse followed by this distance measurement results in a significantly longer procedure compared to the oscillometric measurement method, which in turn increases the cost of PWV assessments conducted by medical assistants. Moreover, the elaborate technique of applanation tonometry is performed by auxiliary personnel who need to be trained, whereas the oscillometric method does not demand investigators to be highly skilled only to position four blood pressure cuffs, one on each limb.

Our results have several clinical implications. Risk stratification is of great importance and recommended by the current guidelines. CAD and PAD are devastating conditions, with substantial impact on patient prognosis, and are associated with increased medical expenditures. Since the PWV can serve as a predictive measure in aged patients and patients with diabetes, end-stage renal disease and ultimately CAD and PAD [26,28–30], a broader implementation could be achieved for automated and time-effective methods.

This study emphasizes that the main advantage of the oscillometric method is the simple handling and faster cfPWV result compared to the conventional tonometric method, without the loss of relevant information. This oscillometric technique detects the arriving pulse wave between the brachial artery and ankle as the baPWV. Although further calculation of the cfPWV is subject to the estimation of the distance between the carotid and femoral arteries, good correlations were found between the two methods.

## Limitations

Data on intraobserver variability are not available. In the context of the present study, it was waived because the oscillometric cfPWV determination is a completely automated procedure. The grouping of CVD-free subjects was based on the known clinical history and assessed ABI. With a mean patient age of 60 years, the indications of some unknown clinical diseases relevant to the PWV in this cohort were not deceptive. Furthermore, due to the concept of the oscillometric detection of the cfPWV, the technique used is not designed for assessment of the augmentation index. The present study investigated the use of only one type of oscillometric device for assessing the PWV. Thus, the results obviously cannot be extrapolated to other available devices, which may include other designated calculation algorithms.

## Conclusion

A simple and rapid automated oscillometric device can provide good diagnostic accuracy in determination of the aortic stiffness through the PWV in both subjects with and without CVD. This time-effective method might help simplify screening for cardiovascular morbidity and atherosclerotic vascular damage, which are major healthcare threats, in daily practice.

## Supporting information

S1 Fig Anonymised data set. (SAV)
